# Pneumonia among under-five children in Alexandria, Egypt: a case-control study

**DOI:** 10.1186/s42506-020-00043-0

**Published:** 2020-07-01

**Authors:** Noha Fadl, Ayat Ashour, Yasmine Yousry Muhammad

**Affiliations:** Department of Family Health, High Institute of Public Health, Alexandria University, Alexandria, Egypt

**Keywords:** Pneumonia, Under-five, Risk factors, Egypt, Case-control study

## Abstract

**Background:**

Pneumonia among under-five children constitutes a major public health concern. Studies examining risk factors for pneumonia in Egypt are limited.

**Aim:**

To identify risk factors of pneumonia among under-five children in Alexandria, Egypt.

**Methods:**

An observational case-control study was conducted over a 5-month period (September 2017–January 2018) in three main pediatric hospitals in Alexandria. A predesigned questionnaire was used to interview 660 mothers of under-five children (330 pneumonia cases and 330 control). The questionnaire included potential risk factors for pneumonia which were categorized into child-related factors, socio-demographic factors, and environmental factors.

**Results:**

The mean age of the children was 23.14 ± 18.61 months with a range of 1–59 months. Nearly two-thirds (58.5%) were boys. Nine factors were found to be independently associated with pneumonia: age ≤ 12 months (OR = 1.925; 95% CI, 1.356–2.733; *P* < 0.001), male gender (OR = 1.646; 95% CI, 1.162–2.332; *P* = 0.005), birth order ≥ 4 (OR = 2.154; 95% CI, 1.282–3.617; *P* = 0.004), low birth weight (OR = 2.562; 95% CI, 1.718–3.821; *P* < 0.001), prematurity (OR = 2.019; 95% CI, 1.154–3.531; *P* = 0.014), place of delivery either at home (OR = 5.669; 95% CI, 1.063–30.237; *P* = 0.042) or at a public hospital (OR = 1.616; 95% CI, 1.131–2.310; *P* = 0.008), presence of concomitant illness (OR = 1.902; 95% CI, 1.043–3.469; *P* = 0.036), poor home aeration (OR = 3.586; 95% CI, 1.971–6.522; *P* < 0.001), and exposure to outdoor air pollution (OR = 2.403; 95% CI, 1.417–4.076; *P* = 0.001).

**Conclusion:**

Several risk factors were significantly related to pneumonia among under-five children. Identifying such factors is important for developing interventions to reduce pneumonia burden among under-five in Egypt.

## Introduction

Pneumonia represents a significant public health problem in the whole world, and it is one of the leading causes of morbidity and mortality among under-five children [1]. Pneumonia is the severe form of acute lower respiratory infections (ALRIs), which nearly kills one million children every year and accounted for 17–19% of all deaths among under-five children. The majority of pneumonia deaths (99%) occur in developing countries [2].

Pneumonia incidence among under-five children is estimated to be 0.05 and 0.29 episodes per child-year in developed and developing countries, respectively. Every year, nearly 156 million new episodes occur worldwide; 151 million episodes are in developing countries, and 7–13% of these episodes are severe enough to be life-threatening and require hospitalization [3].

Evidence has shown a variety of factors that influence pneumonia incidence and severity [4]. The WHO categorized risk factors for pneumonia as (definite, likely, and possible) [5]. Underweight, inadequate breastfeeding, lack of immunization, and indoor and outdoor air pollution are identified as risk factors for childhood pneumonia [6].

Progress in reducing deaths due to pneumonia among under-five children has been significantly slower than for other infectious diseases [7]. For developing effective prevention strategies, a better understanding of the underlying risk factors associated with pneumonia is required. Identifying risk factors or disease determinants is the first step for developing effective prevention strategies. Owing to the scarcity of epidemiological studies in Egypt tackling determinants of pneumonia, this study aims to identify potential risk factors associated with pneumonia among under-five children in Alexandria, Egypt.

## Material and methods

### Study design

A hospital-based, case-control study was conducted over a 5-month period from September 2017 to January 2018.

### Study setting

The study was carried out in three main pediatric hospitals in Alexandria Governorate, Egypt: Al Raml Pediatric Hospital, Al Anfoshy Pediatric Hospital, and Fawzy Moaz General Hospital. These hospitals represent main public hospitals providing low-cost health services to the pediatric population from Alexandria and surrounding governorates.

### Study population

Children aged 1–59 months were enrolled in the study, and we excluded those who had confirmed diagnosis of congenital malformation, tuberculosis, human immunodeficiency virus, sepsis, meningitis, and cardiac and other chronic conditions. Also, those who had cough because of recent history of aspiration of a liquid or of a foreign body and caregivers who refused to participate in the study were also excluded. The cases were children with established diagnosis of pneumonia, which was based on hospital records in which pneumonia was diagnosed according to WHO criteria and radiological findings. Cases were hospitalized in inpatient departments of the sampled hospitals. Controls were apparently healthy children, recruited from out-patient department or hospital’s pharmacy of the same settings of the cases, and did not have previous history or diagnosis of pneumonia.

### Study sample

For sample size calculation, we used Epi-Info, with the following parameters: an estimated rate of 53.2% of exclusively breastfed children in the control group according to World Bank (2010) [8], 90% statistical power to detect an odds ratio (OR) equal to 2.0, with 95% confidence interval and a proportion of 1 case for 1 control. The sample size obtained was 660 patients (330 cases and 330 controls).

### Study tool

To obtain data on potential risk factors, a pre-designed structured questionnaire was used through face-to-face interview with under-five children’s mothers/primary caregivers (supplementary file). The investigated risk factors of pneumonia were among those classified by the WHO [5], as definite (most evidence consistently pointing to the role of the risk factor), likely (most evidence consistently pointing to the role, but with some opposing findings, or scarce but consistent evidence of the role), and possible (with sporadic and inconsistent reports of the role in some contexts).

The questionnaire included (a) child-related factors (age, sex, nutritional status, birth milestones, breastfeeding, vaccination status, and concomitant illness), (b) socio-demographic factors (residence, maternal age, maternal highest educational attainment, maternal occupation, and household crowding index), and (c) environmental factors (indoor and outdoor air pollution). For assessing child’s nutritional status, digital scales appropriate for age were used for measurement of child’s weight.

### Definition of the study variables

- Diagnosis of pneumonia was based on hospital records in which pneumonia was diagnosed according to WHO criteria [9], “cough and/or difficulty breathing with at least one of the following signs: fast breathing, ≥ 50 breaths/minute in a child aged 2–11 months, ≥ 40 breaths/minute in a child aged 12–59 months, or lower chest indrawing” and radiological findings indicative of pneumonia.
- Child’s nutritional status was assessed based on WHO child growth standards weight for age. Children below the 5th percentile were categorized as underweight, meanwhile those plotted above the 95th percentile were considered to be overweight [10].
- Low birth weight is defined by WHO as weight at birth < 2500 g irrespective of gestational age [11].
- Preterm birth is defined by WHO as childbirth before 37 completed weeks of gestation [12].
- Exclusive breastfeeding is defined as “no other food or drink, not even water, except breast milk (including milk expressed or from a wet nurse) for the first 6 months of life but allows the infant to receive oral rehydration solution, drops, and syrups (vitamins, minerals, and medicines)” [13].
- Adolescent mother is defined according to WHO as “those aged 10–19 years” [14].
- Crowding was defined as more than 2 persons per room.
- Indoor air pollution was assessed by exposure to paternal smoke in addition to subjective assessment of home aeration.
- Outdoor air pollution was assessed by presence of a factory near residence.

The questionnaire was revised by an expert pediatrician, and a pilot test of the questionnaire (*n* = 30) was carried out aiming at testing the duration of the interview and wording of the questionnaire.

### Statistical analysis

Collected data was revised for its completeness and accuracy, then coded, computed, and cleaned. Statistical tests were performed using statistical package for social science (SPSS version 21.0). In bivariate analysis, odds ratio with 95% confidence interval (CI) was calculated. Risk factors with a *P* value of ≤ 0.05 in the bivariate analysis were retained in the multivariate analysis. The multivariate analysis was conducted to verify the associations between different independent variables and pneumonia and interpreted as significant at a *P* value of < 0.05 and 95% CI.

## Results

The mean age of the children was 23.14 ± 18.61 months, and nearly two-thirds of them (58.5%) were males. Child-related factors and their association with pneumonia are summarized in Table 1. Children aged ≤ 12 months (OR = 2.254), male gender (OR = 1.783), birth order ≥ 4 (OR = 3.192), low birth weight (OR = 2.688), prematurity (OR = 2.493), place of delivery either at home (OR = 6.971) or at a public hospital (OR = 1.646), inadequate/poor breastfeeding practices (non-breastfed child/not exclusively breastfed child) (OR = 2.060, 1.408), incomplete vaccination (OR = 2.017), and presence of concomitant illness (OR = 2.000) were found to significantly increase the likelihood of pneumonia when compared to the control group.

**Table 1:** Child-related factors associated with the risk of pneumonia among under-five children

Child-related factors	Control (*n* = 330) *N* (%)	Cases (*n* = 330) *N* (%)	*P*	Crude OR	95% CI
**Age**					
1–12 months Older than 12 months	104 (31.5%) 226 (68.5%)	168 (50.9%) 162 (49.1%)	< 0.001*	2.254 1	1.641–3.094
**Sex**					
Boys Girls	170 (51.5%) 160 (48.5%)	216 (65.5%) 114 (34.5%)	< 0.001*	1.783 1	1.304–2.439
**Weight for age**					
Normal Under-weight Overweight	259 (78.5%) 52 (15.8%) 19 (5.7%)	267 (80.9%) 50 (15.2%) 13 (3.9%)	0.268 0.408	1.507 1.405 1	0.729–3.114 0.628–3.144
**Birth order**					
1 2–3 4 +	105 (31.8%) 196 (59.4%) 29 (8.8%)	76 (23.0%) 187 (56.7%) 67 (20.3%)	0.129 < 0.001*	1 1.318 3.192	0.932–1.883 1.886–5.403
**Birthweight** (grams)					
< 2500 ≥ 2500	56 (17.0%) 274 (83.0%)	117 (35.5%) 213 (64.5%)	<0.001*	2.688 1	1.865–3.873
**Prematurity**					
Yes No	25 (7.6%) 305 (92.4%)	56 (17.0%) 274 (83.0%)	< 0.001*	2.493 1	1.514–4.106
**Place of delivery**					
Home Public hospital Private hospital/clinic	2 (0.6%) 183 (55.5%) 145 (43.9%)	10 (3%) 216 (65.5%) 104 (31.5%)	0.013* 0.002*	6.971 1.646 1	1.496–32.483 1.195–2.266
**Mode of birth**					
Caesarian Normal	213 (64.5%) 117 (35.5%)	210 (63.6%) 120 (36.4%)	0.808	1.040 1	0.757–1.430
**Breastfeeding practice**					
No Non-exclusive breastfeeding Exclusive breastfeeding	51 (15.4%) 158 (47.9%) 121 (36.7%)	66 (20.0%) 188 (57.0%) 76 (23.0%)	0.002* < 0.001*	2.060 1.408 1	1.295–3.279 1.152–1.722
**Vaccination status**					
Partially immunized Fully immunized	20 (6.1%) 310 (93.9%)	38 (11.5%) 292 (88.5%)	0.015*	2.017 1	1.147–3.547
**Concomitant illness**					
Yes No	23 (7.0%) 307 (93.0%)	43 (13.0%) 287 (87.0%)	0.011*	2.000 1	1.176–3.402

*OR* odds ratio, *CI* confidence interval

*Statistically significant at *P* ≤ 0.05

Table 2 demonstrates the bivariate analysis for the likelihood of pneumonia based on sociodemographic and environmental factors. Children of an adolescent mother (OR = 3.298), maternal education of ≤ 8 years (OR = 1.495), household crowding index > 2 (OR = 2.689), poor home aeration (OR = 4.259) and exposure to both household cigarette smoke (OR = 1.482), and outdoor air pollution (OR = 3.091) accounted for a significantly higher incidence of pneumonia when compared with control group.

**Table 2:** Sociodemographic and environmental factors associated with the risk of pneumonia among under-five children

Sociodemographic and environmental factors	Control (*n* = 330) N (%)	Cases (*n* = 330) *N* (%)	*P*	Crude OR	95% CI
**Place of residence**					
Rural Urban	48 (14.5%) 282 (85.5%)	64 (19.4%) 266 (80.6%)	0.098	1.414 1	0.938–2.130
**Maternal age** (years)					
≤ 19 20–29 ≥ 30	4 (1.2%) 189 (57.3%) 137 (41.5%)	13 (3.9%) 182 (55.2%) 135 (40.9%)	0.041* 0.885	3.298 0.977 1	1.049–10.37 0.715–1.336
**Maternal education** (years of schooling)					
≤ 8 years > 8 years	155 (47.0%) 175 (53.0%)	188 (57.0%) 142 (43.0%)	0.010*	1.495 1	1.100–2.032
**Maternal occupation**					
Working Housewife	17 (5.2%) 313 (94.8%)	30 (9.1%) 300 (90.9%)	0.052	1.841 1	0.995–3.408
**Household crowding index**					
> 2 0–2	11 (3.3%) 319 (96.7%)	28 (8.5%) 302 (91.5%)	0.007*	2.689 1	1.315–5.496
**Home aeration (subjective)**					
Poor Good	17 (5.2%) 313 (94.8%)	62 (18.8%) 268 (81.2%)	< 0.001*	4.259 1	2.431–7.463
**Household cigarette smoke exposure**					
Yes No	166 (50.3%) 164 (49.7%)	198 (60.0%) 132 (40.0%)	0.012*	1.482 1	1.089–2.017
**Outdoor air pollution** (Factory near residence)					
Yes No	26 (7.9%) 304 (92.1%)	69 (20.9%) 261 (79.1%)	< 0.001*	3.091 1	1.912–4.997

*OR* odds ratio, *CI* confidence interval

*Statistically significant at *P* ≤ 0.05

Based on the findings of the bivariate analysis, 15 variables were introduced for the stepwise multiple logistic regression; age, sex, birth order, birth weight, prematurity, place of delivery, breast feeding, vaccination, concomitant illness, maternal age, education, crowding index, indoor smoking, home ventilation, outdoor pollution. Table 3 shows nine factors independently associated with pneumonia: age ≤ 12 months (OR = 1.925), male gender (OR = 1.646), birth order ≥4 (OR = 2.154), low birth weight (OR = 2.562), prematurity (OR = 2.019), place of delivery either at home (OR = 5.669) or at a public hospital (OR = 1.616), presence of concomitant illness (OR = 1.902), poor home aeration (OR = 3.586), and exposure to outdoor air pollution (OR = 2.403).

**Table 3:** Risk factors independently associated with pneumonia among under-five children (Results of logistic regression)

Variables	Adjusted OR	95% CI	B	S.E.	***P***
**Age ≤ 12 months**	1.925	1.356–2.733	.655	.179	< 0.001*
**Male gender**	1.646	1.162–2.332	.499	.178	0.005*
**Birth order ≥ 4**	2.154	1.282–3.617	.767	.265	0.004*
**Low birth weight < 2500 gm**	2.562	1.718–3.821	.941	.204	< 0.001*
**Prematurity**	2.019	1.154–3.531	.703	.285	0.014*
**Place of delivery:**					
At home At a public hospital	5.669 1.616	1.063–30.237 1.131–2.310	1.735 .480	.854 .182	0.042* 0.008*
**Presence of concomitant illness**	1.902	1.043–3.469	.643	.307	0.036*
**Poor home aeration**	3.586	1.971–6.522	1.277	.305	< 0.001*
**Exposure to outdoor air pollution**	2.403	1.417–4.076	.877	.270	0.001*

*OR* odds ratio, *CI* confidence interval, *B* beta coefficient, *S.E.* standard error

*Statistically significant at *P* ≤ 0.05

## Discussion

In the present study, various factors were reported to be independently associated with pneumonia among under-five children such as younger age, male gender, higher birth order, low birth weight, prematurity, place of delivery, concomitant illness, poor home aeration, and exposure to outdoor air pollution.

Younger children (age of ≤ 12 months) were found to double the likelihood of developing pneumonia when compared to older age group. This finding was in line with other previous studies [15, 16]. This can be explained by the small airways and immature defense system of this age group that make them more susceptible to develop pneumonia. Also, the present study revealed that male gender constituted a significant predictor of acquiring pneumonia in both bivariate and multivariate analysis. In accordance to our finding, a meta-analysis reported that males are more likely than females to acquire pneumonia [17]. Gender variation can be explained by the stronger immune system in girls than boys. There is also evidence that the peripheral airways are narrower during the early years of life in boys, which may predispose them to LRIs [18].

It was further noted that birth order was an important independent variable in bivariate and multivariate analysis; being the 4th child or more increased the likelihood of acquiring pneumonia. Similarly, a recent study reported a relationship between two or more previous pregnancies and pneumonia (2 childbirths: OR = 4.60; ≥ 3 childbirths: OR = 3.25) [19]. Having a large number of children makes the mother pay less attention to her children. It also reflects the household overcrowding which subsequently increases the spread of infection.

Low birth weight was found to be a significant predictor of pneumonia acquisition in bivariate and multivariate analysis. The present finding was supported by findings from a meta-analysis showing that low birth weight was consistently associated with severe LRIs. It showed that the odds ratio meta-estimate was 3.2 (95% CI 1.0–10.0) and 1.8 (95% CI 1.3–2.7) for developing and developed countries, respectively [17]. Barsam et al. also found similar association [20]. Prematurity, was also observed as a significant risk factor in bivariate and multivariate analysis. Similarly, Jackson et al. reported odds ratio to nearly double for premature child [17]. The possible mechanisms that put premature and/or low birth weight child at the risk of respiratory infections are lower immunity and defects in lung function [21].

Delivery at home or in a public hospital was associated with higher risk of pneumonia compared to birth at private hospitals, in both bivariate and multivariate analysis. A previous study conducted in Pakistan supported our finding [22]. Improper implementation of infection control measures may explain our finding.

Lack of breastfeeding is a substantial risk factor of under-five morbidity and mortality in developing countries [23]. Inadequate breastfeeding is significantly associated with pneumonia death (OR = 1.8; 95% CI, 1.2–2.7) [24] and severe pneumonia among under-five children (OR = 2.3; 95% CI, 1.4–3.9) [17]. The breastmilk constitutes a variety of immune-protective and nutritious substances that protect the baby since its own immune system is not mature yet [25].

It is not unexpected that children with comorbidities were twice times more likely to have pneumonia in bivariate and multivariate analysis. In accordance to the present finding, Onyango et al. reported that those who had a comorbidity were more likely to have severe pneumonia (OR = 3.8; 95%CI, 1.4–10.6) [26]. Those who have comorbid diseases may have their immunity lowered making them more vulnerable to severe disease.

Partially immunized children were two times more prone to develop pneumonia compared to up to date/completely immunized children, but this did not last in the final model. When comparing this finding to previous findings, this association was inconsistent. This is probably because *Haemophilus influenzae* type b and pneumococcal conjugate vaccines that are effective in preventing the two most common bacterial causes of childhood pneumonia are not included in the compulsory scheduled vaccination program in Egypt. A meta-analysis did not report any significant association between vaccination status and pneumonia, which supported the present finding [17]. On the other hand, two studies found an association between incomplete immunization and severe ALRI [27, 28].

Mother’s education was inversely related to pneumonia but these associations failed to persist in the final model. Similarly, a recent meta-analysis found that the relationship between the lack of maternal education and severe respiratory infection was inconsistent [17]. Other factors rather than maternal education such as previous experience of the illness could affect mothers’ health behavior and thus incidence of pneumonia.

Another socio-demographic factor emerged in the current study was overcrowding (crowding index > 2). However, it was surprisingly no longer present in the final model. This was in contradictory to a previous study [17]. It may reflect the complex intertwining association between socioeconomic factors and health.

In terms of environemntal factors, poor home aeration was significantly associated with increasing risk of pneumonia, and it persisted in the final model. Our result was consistent with findings of Zheng et al. [29]. Also, it was found that child exposure to a household cigarette smoke increased acquiring pneumonia by 1.5 folds; however, this association was not retained in the final model. A previous study similarly showed an inconsistent association between exposure to cigarette smoke and pneumonia acquisition [17]. On the other hand, exposure to parental smoking was found to increase the likelihood of severe childhood pneumonia [30] and was an independent risk factor of poor outcome in another study (OR = 1.8; 95% CI, 1.1–4.1) [31]. Additionally, exposure to outdoor air pollution was a significant variable in both bivariate and multivariate analysis. Similarly, the impact of outdoor air pollution on child health was of a particular concern in regions with traffic congestion and industrial air pollution. Exposure to greater levels of nitrogen dioxide produced by vehicle traffic was associated with the likelihood of pneumonia occurrence among under-five children (OR = 1.1; 95% CI, 0.6–2.2 and OR = 3.3; 95% CI, 1.3–8.1 respectively) after adjustment of cigarette exposure [32].

### Limitations of the study

The data collected in the current study was obtained by face-to-face interview questionnaire which is subjective. Additionally, some variables were self-reported as concomitant illness and child’s birth weight which might be inaccurate or introduce recall bias.

## Conclusion

Since pneumonia is still ranked among the top causes of child morbidity and mortality, effective strategies and interventions to reduce the incidence of pneumonia should not be ignored especially in developing countries. Identifying risk factors and quantifying the strength of their association with the disease could guide such strategies and interventions. Several risk factors were found to be associated with pneumonia in this study, and the following are recommended to address these factors; improving antenatal care could help to decrease low birth weight, prematurity, and help mothers to adopt suitable plans of birth which in turn could decrease the rate of home deliveries. Also, antenatal care could be considered as entry point for raising mother’s awareness about appropriate feeding practice including the importance of exclusive breast feeding. Strengthening family planning services to reduce overcrowding as well as conducting health education campaigns to raise awareness about risk factors of the disease especially indoor and outdoor pollution and preventive measures might help to reduce pneumonia morbidity and mortality among under-five.

## Supplementary information

**Additional file 1.** Data Collection Tool.

## Data Availability

Data will be available on reasonable request. Please contact the corresponding author for data requests.

## Abbreviations

ALRIs: Acute lower respiratory infections
CI: Confidence interval
OR: Odds ratio
WHO: World Health Organization
