# Supplementary material for: Pneumonia among under-five children in Alexandria, Egypt: a case-control study
Source: J Egypt Public Health Assoc. 2020 Jul 1;95:14. doi: 10.1186/s42506-020-00043-0 (PMC7364693; doi:10.1186/s42506-020-00043-0)
Supplement: Supplementary file 1 — Additional file 1. Data Collection Tool. [file 42506_2020_43_MOESM1_ESM.docx]

**Data collection tool** **for mothers/ primary caregivers of under five children with pneumonia**

**- Serial number: - Date: - Name of hospital: - Diagnosis:**

1. **Socio-demographic data**
2. Place of residence: 1) Urban 2) Rural
3. Age of the mother: ………..
4. Education of mother: 1) Illiterate / read and write 2) Primary 3) Preparatory 4) Secondary 4) University
5. Total years of schooling of mother: ………..
6. Working status of mother: 1) Working 2) Housewife
7. Family size: ………..
8. Number of sleeping rooms at home: ………..
9. Crowding index: ………..
10. **Environmental characteristics**
11. Paternal smoking in home: 1) Yes 2) No
12. Ventilation of the home: 1) Good 2) Bad
13. Factory near residence: 1) Yes 2) No
14. **Child’s characteristics**
15. Child’s Age in months: ………
16. Sex: 1) Male 2) Female
17. Weight of child (in Kilograms): ………
18. Weight for age: 1) Normal 2) Underweight 3) Overweight
19. Birth order: 1) 1 2) 2-3 4) 4-5 5) 6+
20. Weight at birth (in grams): 1) < 2500 2) ≥ 2500
21. Premature labor (< 37 gestational week): 1) Yes 2) No
22. Place of birth: 1) Home 2) Private hospital/ clinic 3) General hospital 4) Others (mention)
23. Type of delivery: 1) Normal 2) Cesarean section
24. Did you breastfeed your baby? 1)Yes 2) No
25. If the answer to previous question yes, mention duration of breastfeeding in months: …….
26. Introducing anything in addition to breastfeeding during first 6 months of child? 1) No 2) Yes (mention)
27. Child’s immunization history: 1) Fully immunized 2) Partially immunized 3) Not immunized at all
28. Mention other health concomitant condition: ………..

المعهد العالي للصحة العامة

منظمة الصحة العالمية

المكتب الاقليمي

جامعة الأسكندرية

**استبيان لدراسة عوامل الخطر المصاحبة للإلتهاب الرئوى بين الأطفال دون سن الخامسة فى الإسكندرية**

- **التاريخ: - اسم المستشفى:**
- **الرقم المسلسل: - التشخيص:**

| **بيانات خاصة بالطفل:** | | | **الكود** | |
| --- | --- | --- | --- | --- |
| **عمر الطفل بالأشهر** | | **………** |  | |
| 1. **الجنس** | | **(1) ذكر (2) أنثى** |  | |
| 1. **وزن الطفل (بالكيلوجرام)** | | **………** |  | |
| 1. **الوزن بالنسبة للعمر** | | **(1) طبيعي (2) نقص الوزن (3) زيادة الوزن** |  | |
| 1. **وزن الطفل عند الولادة (بالجرام) ؟** | | **(1) >2500 (2) ≥2500** |  | |
| 1. **مكان الولادة كان فين؟** | | **(1) البيت (2) مستشفى حكومى (3) مستشفى خاصة 4) عيادة خاصة (5) اخرى (تذكر)** |  | |
| 1. **نوع الولادة؟** | | **(1) طبيعى (2) قيصرى** |  | |
| 1. **هل كانت الولادة مبكرة (اقل من الاسبوع37)؟**   (هل دخل حضانة واتحطله اكسجين؟) | | **(0) لا (1) نعم** |  | |
| 1. **هل رضعتى ابنك رضاعة طبيعية ؟** 2. لو رضعتى طبيعى قد ايه مدة الرضاعة الطبيعية؟ (بالشهور) 3. هل دخلتى اى حاجة مع الرضاعة الطبيعية اول ست شهور من عمر الطفل؟ | | **(0)لا (اذهب لسؤال 12) (1)نعم**  **..........**  (0)لا (1) لبن صناعى (2)لبن بقرى (3)اعشاب (4)مياه شرب (5) أخرى (تذكر)....... |  | |
| 1. **هل استكمل الطفل التطعيمات حسب العمر؟** | | **(1) نعم كلها (2) جزء منها (3) لا لم يأخذ اى تطعيم** |  | |
| 1. **ترتيب الطفل بين اخواته** | | **(1) 1 (2) 2-3 (3) 4-5 4) 6+** |  | |
| 1. **هل الطفل عنده أى مشاكل صحية أخرى (حادة أو مزمنة) ؟** | | **(0) لا (1) نعم تذكر ........** |  | |
| **بيانات اجتماعية:** | | | | |
| 1. **مكان الإقامة** | **(1) الحضر (2) الريف** | | |  |
| 1. **سن الأم** | **……….** | | |  |
| 1. **تعليم الأم** | **(1) غير متعلمة /تقرا وتكتب (2) الابتدائية (3) الاعدادية (4) الثانوية/ دبلوم (5) جامعى** | | |  |
| 1. **مجموع سنوات الدراسة؟** | **……….** | | |  |
| 1. **وظيفة الام** | **(0) لا تعمل (1) تعمل** | | |  |
| 1. **عدد أفراد الأسرة** | **……….** | | |  |
| 1. **عدد غرف المنزل +الصالة** | **……….** | | |  |
| 1. **معدل الازدحام** | **……….** | | |  |
| **بيانات خاصة بالسكن:** | | | | |
| 1. **هل الوالد بيدخن؟** | **(0) لا (1) نعم** | | |  |
| 1. **هل التهوية جيدة فى البيت؟** | **(0) لا (1) نعم** | | |  |
| 1. **هل فى مصنع قرب السكن؟** | **(0) لا (1) نعم** | | |  |
